# An Unusual Experience in Practice

**Published:** 1899-10

**Authors:** I. B. Howell

**Affiliations:** Paducah, Ky.


ARTICLE XI.

### AN UNUSUAL EXPERIENCE IN PRACTICE.

I. B. HOWELL, D. D. S., PADUCAH, KY.

It is difficult to define an unusual experience in practice, or to select an occurrence in one's own experience that may not have occurred frequantly in the experience of others.

I have selected a case in practice, however, which stands isolated in my own short experience, and yet one that has given me considerable thought, not to say some little anxiety, and which possibly has not occurred at all in the experience of many who are here present. On the morning of June 1st, a man about twenty-five years old came into my office suffering intensely with pain at the inside angle of the lower jaw on the left side and radiating over that entire side of the face and neck.

There was considerable inflammation and swelling and extreme sensitiveness of the parts, and a degree of tonic spasms of the muscles of that side that no reasonable amount of force could overcome. My diagnosis was, a belated, developing third molar, trying to make its way into a space already impinged upon by the second molar, and hence inadequate to the reception of the third molar, with possibly adjoining decayed teeth, periostitis, mastitis and an element of reflex muscular spasm.

Calling a physician near by to administer chloroform, I had the patient, with his consent, anesthetized, and then, partially prying open the mouth, I found the second molar badly decayed and the third molar, or wisdom tooth, tightly wedged in and occupying a horizontal position between the second molar and vertical ramus of the jaw.

Believing the case to be one that demanded immediate relief, the third molar being difficult if not impossible to get at without an unwarranted degree of violence to inflamed tissues, and the second molar, being already decayed and practically worthless, I extracted the latter intending to then extract the misplaced third molar also. But just at this juncture the patient passed from under the influence of chloroform and stubbornly rebelled against any further inhalation of the anesthetic or interference with his mouth whatever.

Thinking that perhaps enough had been done to relieve the intense pain and to allow the inflammation to subside, I ceased all efforts at further interference and instructed the patient to call later for additional treatment.

Now, perhaps there is nothing unusual in all of this. Were I to stop just here this paper would certainly amount to little else than a blank cartridge.

But the unusual part of the experience to me, and perhaps would have been to most of you, was that the patient, instead of calling again for further treatment, instituted a suit in court for $1,000 on the plea that I had extracted the wrong tooth.

This proceeding not only made the experience unusual to me but interesting as well. It made necessary the cultivation of the acquaintance of a gentleman in another profession —a lawyer.

It was for the purpose of carrying me into the presence of a judge and jury and to have men wholly unacquainted with the requirements and duties of my profession pass upon the merits or demerits of my practice in an emergency.

It was to make me a representative before the court of all the members of my profession in this country with all their vast interest, and to establish a precedent in medico-legal jurisprudence that would tend to make dentists mere mechanical imitators of what some one else had already done and published on the one hand, or intelligent, independent operators in oral surgery on the other hand.

Hence, this case of unusual experience in the practice affects the rights and involves the interests of us all.

And I am proud to be able to say that a mere knowledge of its existence has aroused the professional patriotism of the rank and file, as well as the leading lights of our honorable profession, wherever such facts have been made known.

While I will not say that I believe I could call to my aid in the contest twelve legions of dentists, I will say that I believe I could call to my aid every honest dentist in these United States, and I believe further. that most of them are honest.

It may be of interest to you to know that the lawsuit has never been tried. The plantiff put it off from court to court, seeking in the meantime a compromise on the basis of a small money consideration from me, until recently he took pneumonia and died, and his widow then sent to know if I would pay her a small consideration to withdraw the suit.

I feel and have all the time felt that to compromise this matter by the payment of one single penny was to compromise myself and my profession, and hence it has been and is now my motto to fight it out from Cape Cod to Kalamazoo on the line of honorable defense, let it cost what it may.—*Indiana Dental Journal.*

